# Baseline Plasma Metabotype Correlates With Direct-Acting Antiviral Therapy Nonresponse for HCV in HIV–HCV Coinfected Patients

**DOI:** 10.3389/fmolb.2021.748014

**Published:** 2022-01-10

**Authors:** Gaurav Tripathi, Sheetalnath Rooge, Manisha Yadav, Babu Mathew, Nupur Sharma, Vasundhra Bindal, Hamed Hemati, Jaswinder Singh Maras, Ekta Gupta

**Affiliations:** ^1^ Department of Molecular and Cellular Medicine, Institute of Liver and Biliary Sciences, New Delhi, India; ^2^ Department of Clinical Virology, Institute of Liver and Biliary Sciences, New Delhi, India

**Keywords:** metabolomic analyses, HCV (hepatitis C), HIV - human immunodeficiency virus, coinfection (HIV infection), direct-acting antiviral drugs

## Abstract

**Introduction:** With the advent of direct-acting antiviral (DAA) therapy for HCV, the cure is achieved at similar rates among HIV–HCV coinfected patients as in HCV mono-infected patients. The present study evaluates host plasma metabolites as putative indicators in predicting the treatment response in baseline HIV–HCV patients.

**Methods:** Non-cirrhotic HIV–HCV (N = 43) coinfected patients were treated with sofosbuvir and daclatasvir for 12 weeks. Plasma metabolite profiling of pre- and post-therapy was analyzed in 20/43 patients. Of the 20 selected, 10 (50%) attained the sustained viral response [(SVR) (responders)] as defined by the absence of HCV RNA at 12 weeks after the treatment, and 10 (50%) did not attain the cure for HCV (nonresponders).

**Results:** A total of 563 features were annotated (metabolomic/spectral databases). Before therapy, 39 metabolites differentiated (FC ±1.5, *p* < 0.05) nonresponders from responders. Of these, 20 upregulated and 19 downregulated were associated with tryptophan metabolism, nicotinamide metabolism, and others. Post therapy, 62 plasma metabolites (12 upregulated and 50 downregulated, FC±1.5, *p* < 0.05) differentiated nonresponders from responders and highlighted a significant increase in the steroid and histidine metabolism and significant decrease in tryptophan metabolism and ascorbate and pyruvate metabolism in the nonresponders. Based on random forest and multivariate linear regression analysis, the baseline level of N-acetylspermidine (FC > 2, AUC = 0.940, Bfactor = −0.267) and 2-acetolactate (FC > 2, AUC = 0.880, Bfactor = −0.713) significantly differentiated between nonresponders from responders in HIV–HCV coinfected patients and was able to predict the failure of treatment response.

**Conclusion:** Increased baseline levels of N-acetylspermidine and 2-acetolactate levels are associated with the likeliness of failure to attain the cure for HCV in HIV–HCV coinfected patients.

## Introduction

According to UNAIDS and WHO, 38.0 million (31.6 million–44.5 million) people are currently living with an HIV infection globally. Among these patients, 2–15% of patients are coinfected with an additional HCV infection (Global HIV & AIDS Statistics; Global hepatitis report, 2017). The HIV-infected patients are more prone to acquire an HCV coinfection because both of these infections are blood borne and share a similar transmission route (Victoria et al., 2010). The significant population that harbors HIV–HCV coinfection includes patients such as hemophiliacs, men who had unprotected sex with other males (MSM), and patients with intravenous drug abuse (PWID) (Pineda et al., 2015). Compared to HCV mono-infected patients, HIV–HCV coinfected patients have relatively three times fast progression toward liver fibrosis and are thus more prone to liver cirrhosis, hepatocellular carcinoma, and antiretroviral therapy–mediated hepatotoxicity (ATMH) (Baranoski et al., 2015).

Initially, pegylated interferon (PEG-IFN) and ribavirin (RBV) were used to treat chronic HCV infection, but a low SVR attainment rate was the primary concern (Palumbo, 2011). This led to the discovery of direct-acting antivirals (DAAs). In 2011, first DAAs, telaprevir and boceprevir, were approved to treat HCV genotype-1 infection (Murphy et al., 2014). These drugs were NS3/4A protease inhibitors and showed improved SVR rates to 75–80% when administered together with PEG-IFN and RBV. But still, complicated dosing and co-interaction with other antiviral drugs were the primary concern (Bichoupan et al., 2017). In 2014, new DAAs were approved belonging to different classes such as NS3/4A protease inhibitors, NS5A replication complex inhibitors, nucleoside, and non-nucleoside polymerase inhibitors. Their fixed dosage compensation was also equilibrated, ultimately increasing the SVR attainment rate to 90% (Bailly et al., 2015). But still, there is an HIV–HCV population that includes DAA nonresponders, and thus these patients still have a higher mortality rate (Patel et al., 2020).

Currently, no indicators correlate with the prediction of DAA therapy in response to HIV–HCV coinfected patients (Debnath et al., 2020). Thus, in this study, we hypothesize that those HIV–HCV patients who are DAA responders have a distinct metabolic signature compared to those who are non-responders. The primary aim of the present study was to identify baseline metabolites that could identify potential HIV–HCV coinfected nonresponders of the DAA therapy. These distinct metabolic signatures can provide pathophysiological insights that can develop new diagnostic methods even before starting the therapy. We designed a study in which HIV–-HCV coinfected patients were enrolled and subjected to DAA therapy. These patients were administered with sofosbuvir, an NS3/4A protease inhibitor, and daclatasvir, an NS5A replication complex inhibitor, for a brief period of 3 months (Smith et al., 2016). Post therapy, HCV viral titer was checked, and patients tested HCV-negative were considered DAA responders, while those tested positive were considered DAA nonresponders. In a pilot study setting, we performed the global untargeted metabolomics on the plasma samples of HIV–HCV coinfected DAA responders and nonresponders at baseline and post 3 months of DAA therapy to test our hypothesis. This was followed by pathway enrichment analysis which could provide insight into the pathophysiology and classification of baseline indicators of response to DAA therapy in HIV–HCV coinfected patients.

## Patients and Methods

In this retrospective pilot study, patients infected with HCV (*n* = 2,049) were seen at the Department of Virology, Institute of Liver and Biliary Sciences, New Delhi, India, and confirmed to have HIV and HCV coinfection via measuring IgG serum cutoff for HIV and HCV, and HCV viral titer and genotype assessment at baseline were identified and included in the study (*n* = 43; 2%). These patients were treated using DAA therapy for 8 weeks, and written informed consent was obtained from all patients before starting therapy. About 12 weeks post therapy, HIV–HCV coinfected patients were again tested for HCV viral load, and patients negative for viral antigen were referred to as DAA responders. Patients still positive for viral antigen were referred to as DAA nonresponders. One patient died during treatment, eight did not complete DAA therapy, 14 were lost to follow up, and only 20 completed the DAA therapy. Of the 20 patients, 10 patients (50.0%) positively responded to DAA therapy, while 10 (50.0%) were nonresponders. Only these 20 patients were included in the baseline or post-therapy metabolome profile analysis and correlation to DAA therapy response (Supplementary Figure S1) (Note that all the sample processing and handling were performed in a BSL-3 facility established at the Department of Virology, Institute of Liver and Biliary Sciences, New Delhi, India).

### Protocol of Plasma Metabolome Processing

An organic phase extraction protocol was used to isolate metabolites from 50 µl of the plasma sample (Salem et al., 2017; Wawrzyniak et al., 2018). About 450 µl methanol was added to 50 µl of the plasma sample and kept overnight at −20°C for protein precipitation. These samples were centrifuged at 13,000 rpm for 10 min. The protein pellet was discarded, and the supernatant was isolated, dried under vacuum conditions and reconstituted with (90:5:5) 90% water:5% internal standards:5% acetonitrile and then loaded for reverse-phase chromatography in a C18 column (Thermo Scientific™25003102130: 3 μm, 2.1 mm, and 100 mm) using an ultra-high-performance liquid chromatographic system followed by high-resolution orbitrap mass spectrometry (HRMS) (Chekmeneva et al., 2018). Compound Discoverer 3.0 was used to identify metabolite features (ThermoFisher Scientific, Waltham, United States) (Chekmeneva et al., 2018). The features were annotated using mass list (Chekmeneva et al., 2018; Boudah et al., 2014) and mzCloud™ (www.mzcloud.org). Identified and annotated features were subjected to log normalization and Pareto-scaling using a MetaboAnalyst 5.0 (http://metaboanalyst.ca.) server (Torres et al., 2019) and used to perform principal component analysis and partial least square discriminant analysis, heat maps, random forest analysis, and others. Pathway enrichment patterns were analyzed using MetaboAnalyst 5.0 (Torres et al., 2019). IBM SPSS Statistics version 26 was used for multivariate, correlation, and univariate regression analysis.

### Statistical Analyses

The results are shown as mean ± SD unless indicated otherwise. Using GraphPad Prism v6, statistical analyses were performed, and *p*-values of <0.05 were considered statistically significant. To compare variables between the two groups, the unpaired (two-tailed) Student’s *t*-test and Mann–Whitney test were performed. For comparison among more than two groups, one-way ANOVA, Kruskal–Wallis test, was performed. Adjusted *p* values and log fold change in mass spectrometry analysis (LC-MS/MS) data were calculated according to Benjamini and Hochberg. Expression in terms of metabolome fold changes was computed based on intensity values. Functional analysis was performed with MetaboAnalyst and KEGG pathway enrichment. All correlations were performed using Spearman correlation analysis. Univariate and multivariate linear regression analysis was performed using SPSS 20.

## Results

### Baseline Demographic Profile of the Study Population

In a pilot study setting, a total of 2,049 (*n* = 2,049) HCV patients were screened for HIV coinfection. In our study, 2% (*n* = 43) patients were identified as HIV–HCV coinfected patients. These patients received sofosbuvir and daclatasvir as standard HCV therapy. Amongst them, 20 paired patient samples were identified, and their baseline and post-therapy (3 months) plasma samples were subjected to metabolomic evaluations. Based on the post-therapy HCV RNA status, these patients were further segregated as responders (*n* = 10) and nonresponders (*n* = 10) to standard HCV therapy (Supplementary Figure S1). The baseline clinical and the demographic profile of the study population are shown in Supplementary Table S1. Clinical parameters of nonresponders were comparable to those of responders (*p* < 0.05).

### Baseline Plasma Metabolome Profile Robustly Segregates DAA Nonresponders From DAA Responders

**FIGURE 1:**
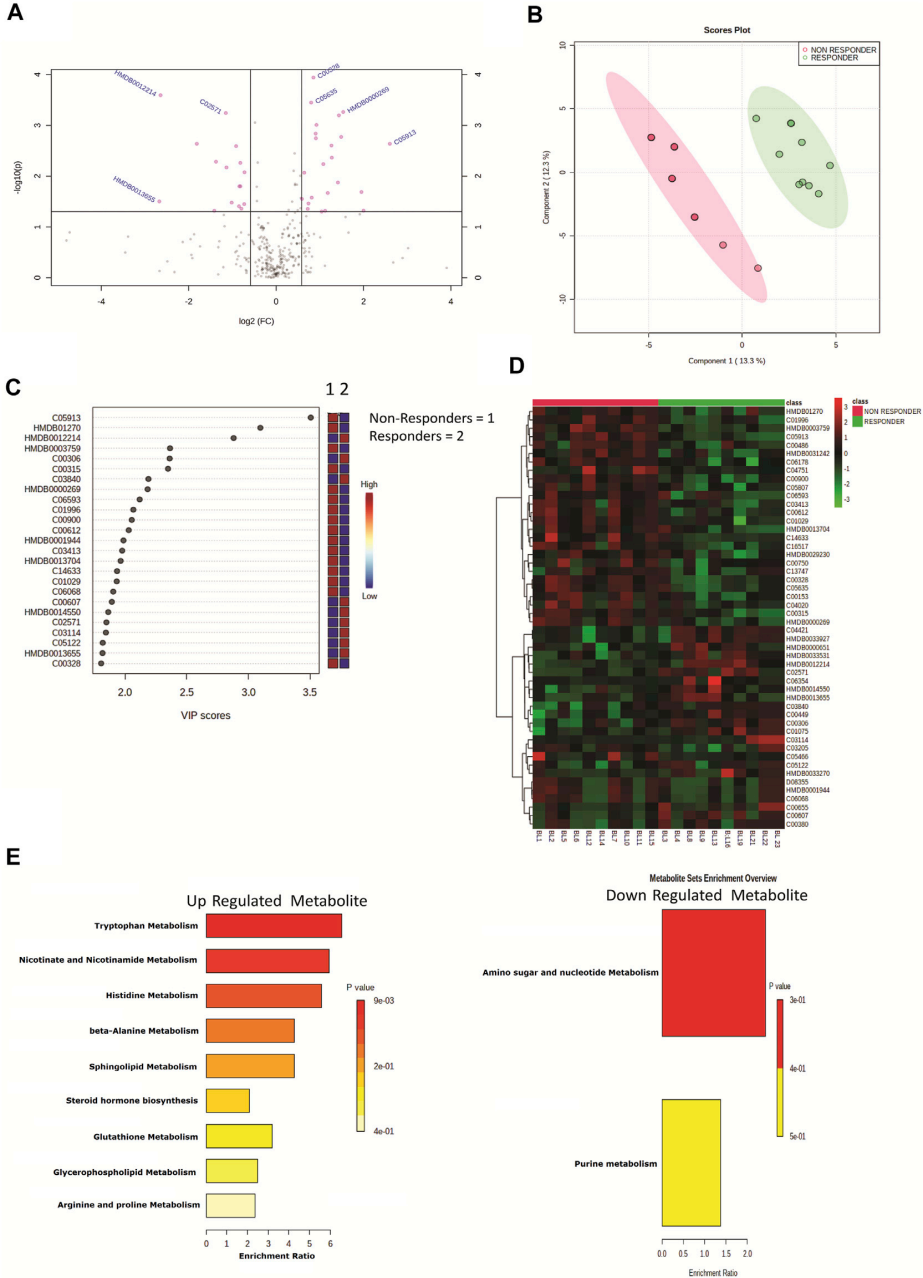
**(A)** Volcano plot showing differentially expressed metabolites in DAA nonresponders vs. DAA responders at baseline. Pink dots are significant at *p* < 0.05. **(B)** Partial least square discriminant analysis (PLSDA) showing clear segregation of DAA nonresponders and DAA responders at baseline based on the metabolome profile of patients. Pink dots correspond to nonresponders, and green dots correspond to responders. **(C)** Variable importance in projection (VIP) plot displaying the top 20 most important metabolite features identified by PLSDA at baseline. Colored boxes on the right indicate relative concentration of the corresponding metabolite between DAA nonresponders vs. DAA responders. **(D)** Heat map and hierarchical clustering analysis of top 50 metabolites are capable to segregate DAA nonresponders (red bar) from DAA responders (green bar) at baseline. The expression is given as red = upregulated, green = downregulated, and black = unregulated. **(E)** KEGG pathway analysis of upregulated and downregulated metabolites in DAA nonresponders as compared to those in DAA responders at baseline.

Plasma samples of HIV–HCV coinfected patients at baseline and post therapy (3 months) were subjected to an untargeted metabolomic evaluation. A total of 10,905 features were identified in a positive and negative ionization mode. Of those, 563 features were annotated and were compared between the study groups. Baseline evaluation of the metabolome profile between the responders and nonresponders resulted in the identification of 39 differentially expressed metabolites (DEMs; FC > 1.5, *p* < 0.05) (Figure 1A, Table 1). Of those, 20 were significantly upregulated, and 19 were downregulated (Figure 1A, Table 1). The partial least square discriminant analysis along with hierarchical clustering analysis was able to completely differentiate DAA nonresponders from responders (Figures 1B,D) and provide a list of the most important metabolite (VIP; variable importance in projection) features (Figure 1C). The metabolites upregulated in nonresponders were associated with tryptophan metabolism, nicotinamide metabolism, and others (Figure 1E). Similarly, downregulated metabolites were associated with amino sugar and nucleotide sugar pathway in DAA nonresponders compared to responders Figure 1E). These results signify that the baseline metabolome profile of DAA nonresponders differs from that of DAA responders, and an increase in tryptophan metabolism and linked pathways could be exploited to identify candidate indicators of DAA response in HIV–HCV coinfected patients.

**TABLE 1:** Differentially expressed metabolites in DAA Nonresponders compared to responders at baseline and at post therapy [significance (*p* < 0.05)].

Baseline (Nonresponders vs. responders)						Post therapy (Non responders vs. responders)								
Upregulated			Downregulated			Upregulated			Downregulated					
KEGG or HMDB ID	FC	*p* value	KEGG or HMDB ID	FC	*p* value	KEGG or HMDB ID	FC	*p* value	KEGG or HMDB ID	FC	*p* value	KEGG or HMDB ID	FC	*p* value
C05913	6.1	0.01	C02214	1.7	0.01	HMDB0029230	5.0	0.05	C15976	0.7	0.04	C16759	0.4	0.02
HMDB0001944	4.0	0.03	HMDB0033531	1.7	0.03	C06068	4.2	0.01	HMDB001З817	0.7	0.04	C00607	0.4	0.02
C14633	3.9	0.01	C00666	1.7	0.03	HMDB0001004	3.7	0.05	C00900	0.7	0.04	HMDB0040344	0.4	0.03
HMDB0000269	2.9	0.01	HMDB0033927	1.8	0.01	HMDB0001944	3.5	0.01	C04З68	0.7	0.05	C00170	0.4	0.02
HMDB0003759	2.8	0.01	C00449	1.8	0.04	C08355	3.2	0.01	C00819	0.6	0.01	HMDB0002577	0.4	0.02
C00315	2.7	0.01	C04421	1.8	0.01	HMDB0029378	3.2	0.05	C01746	0.6	0.02	HMDB000706	0.4	0.01
C03413	2.7	0.01	HMDB0000651	1.8	0.05	C05793	2.9	0.03	C01037	0.6	0.01	C05905	0.3	0.04
C00153	2.4	0.01	C01075	1.9	0.01	HMDB0003759	2.5	0.02	HMDB0006050	0.6	0.04	C01234	0.3	0.01
C00612	2.4	0.01	C03840	2.2	0.03	C04081	2.1	0.01	C04303	0.6	0.03	C02354	0.3	0.01
HMDB01270	2.2	0.05	C02571	2.2	0.01	CО6196	1.9	0.04	C02855	0.6	0.01	C11504	0.3	0.01
C01029	2.1	0.01	HMDB0014550	2.6	0.01	C05771	1.8	0.01	C01772	0.6	0.02	C00380	0.3	0.01
C00900	1.9	0.01	C00306	3.5	0.01	C05827	1.8	0.01	C08493	0.6	0.03	C06087	0.3	0.01
C01996	1.9	0.01	HMDB0012214	6.3	0.01				HMDB0029493	0.6	0.04	C07481	0.3	0.01
C16517	1.9	0.01	HMDB0013655	6.4	0.04				C08313	0.6	0.02	HMDB0001043	0.3	0.36
C00328	1.8	0.01	HMDB0029493	0.6	0.05				C06960	0.6	0.04	C00306	0.2	0.01
C05635	1.8	0.01	C06593	0.6	0.05				C09715	0.6	0.01	HMDB0001390	0.2	0.05
C05807	1.7	0.05	C00477	1.8	0.05				HMDB0013713	0.5	0.04	C00655	0.1	0.01
C06178	1.6	0.02	C03665	6.4	0.05				C08255	0.5	0.05			
C05828	1.5	0.04							C00463	0.5	0.01			
									C0025б	0.5	0.02			
									C01796	0.5	0.04			
									C00137	0.5	0.02			
									C01075	0.5	0.02			
									C00078	0.5	0.04			
									C19463	0.5	0.04			
									C00643	0.5	0.04			
									C02572	0.5	0.01			
									HMDB0000734	0.5	0.04			
									C15967	0.5	0.04			
									C00644	0.5	0.01			
									C04503	0.5	0.01			
									C06323	0.5	0.01			
									HMDB0010З61	0.4	0.04			

### Post-DAA Therapy Plasma Metabolome Profile Also Differentiates DAA Nonresponders

**FIGURE 2:**
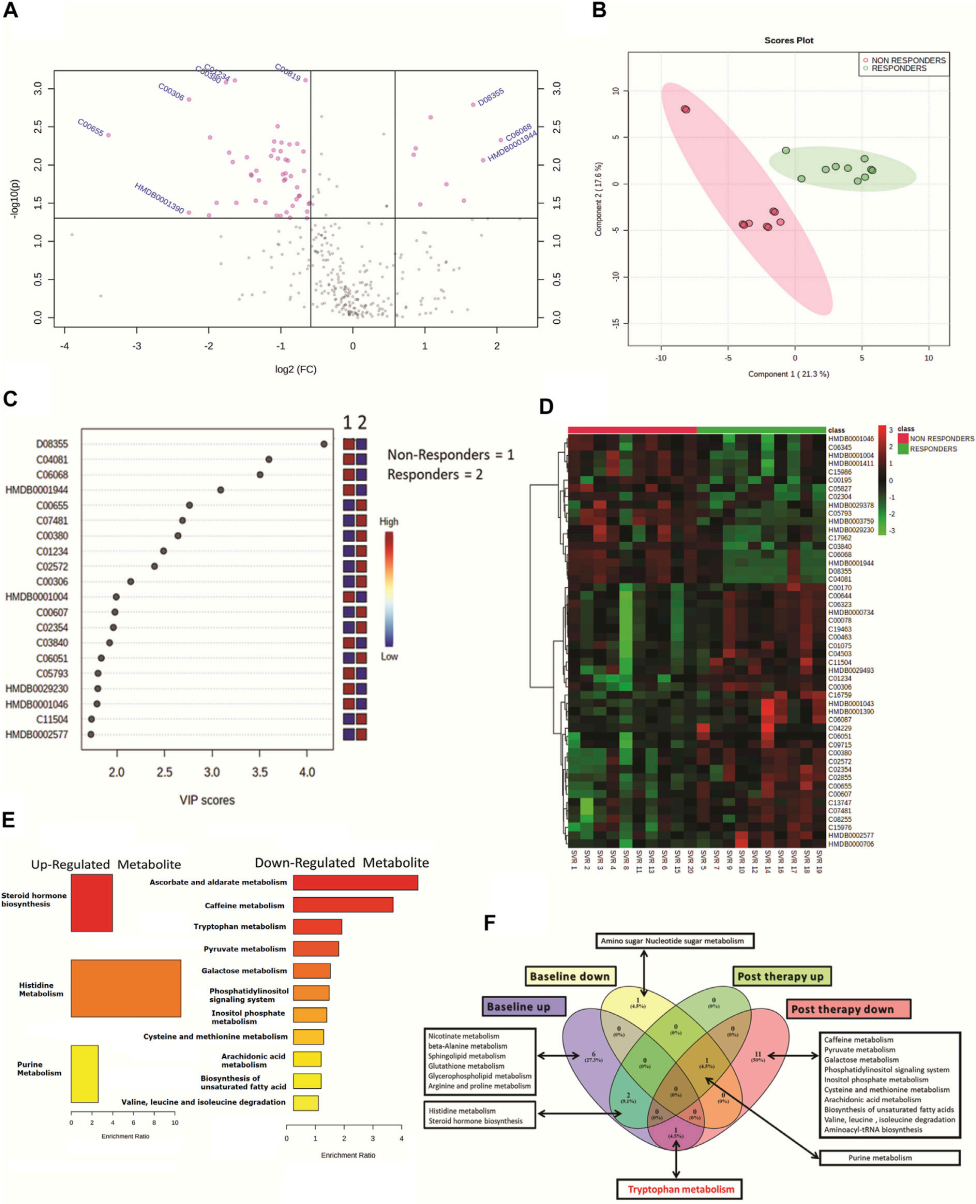
**(A)** Volcano plot showing differentially expressed metabolites in DAA nonresponders vs. DAA responders post DAA therapy. Pink dots are significant at *p* < 0.05. **(B)** Partial least square discriminant analysis (PLSDA) showing clear segregation of DAA nonresponders and DAA responders post therapy based on the metabolome profile of patients. Pink dots correspond to nonresponders, and green dots correspond to responders. **(C)** Variable importance in projection (VIP) plot displaying the top 20 most important metabolite features identified by PLSDA post therapy. Colored boxes on the right indicate relative concentration of the corresponding metabolite between DAA nonresponders vs. DAA responders post therapy. **(D)** Heat map and hierarchical clustering analysis of top 50 metabolites are capable to segregate DAA nonresponders (red bar) from DAA responders (green bar) post therapy. The expression is given as red = upregulated, green = downregulated, and black = unregulated. **(E)** KEGG pathway analysis of upregulated and downregulated metabolites in DAA nonresponders as compared to those in DAA responders post therapy. **(F)** Venn diagram showing unique and common pathways in nonresponders vs. responders at baseline as well as post therapy.

Post-DAA therapy plasma metabolome profiles of DAA nonresponders were compared to those of DAA responders. A total of 62 differentially expressed metabolites (DEMs) were identified (FC > 1.5, *p* < 0.05) (Figure 2A, Table 1). Of those, 12 were significantly upregulated, and 50 were downregulated in DAA nonresponders compared to those of responders (Table 1). Partial least square discriminant analysis and hierarchical clustering analysis significantly differentiated between DAA nonresponders and responders based on the post-therapy metabotype (Figures 2B,D) and identified the most important metabolite (VIP) features shown in Figure 2C. Post DAA therapy metabolites significantly upregulated in nonresponders were linked with steroid hormone biosynthesis, histidine metabolism, and purine metabolism (Figure 1E). Similarly, metabolites downregulated were associated with ascorbate metabolism, tryptophan metabolism, pyruvate metabolism, etc. (Figure 1E). Comparative analysis of the baseline and post-therapy metabolome profile of DAA nonresponders highlighted that DAA nonresponders at baseline uniquely regulated an increase in nicotinate metabolism, beta-alanine metabolism, sphingolipid metabolism, glutathione metabolism, glycerophospholipids metabolism, and arginine and proline metabolism. Whereas DAA nonresponders post therapy uniquely decreased metabolites associated with ascorbate metabolism, tryptophan metabolism, pyruvate metabolism, and other pathways. Interestingly, DAA therapy significantly reduced tryptophan metabolism and associated metabolites in nonresponders compared to those in responders. These observations suggest that the post-therapy metabolome profile is distinct for nonresponders. A decrease in tryptophan metabolism and associated metabolites post therapy could be associated with increased utilization of polyamines during active HCV replication seen in DAA nonresponders (Figure 1F).

### HCV Genotype and Its Association With Baseline and Post-Therapy Metabotype

**FIGURE 3:**
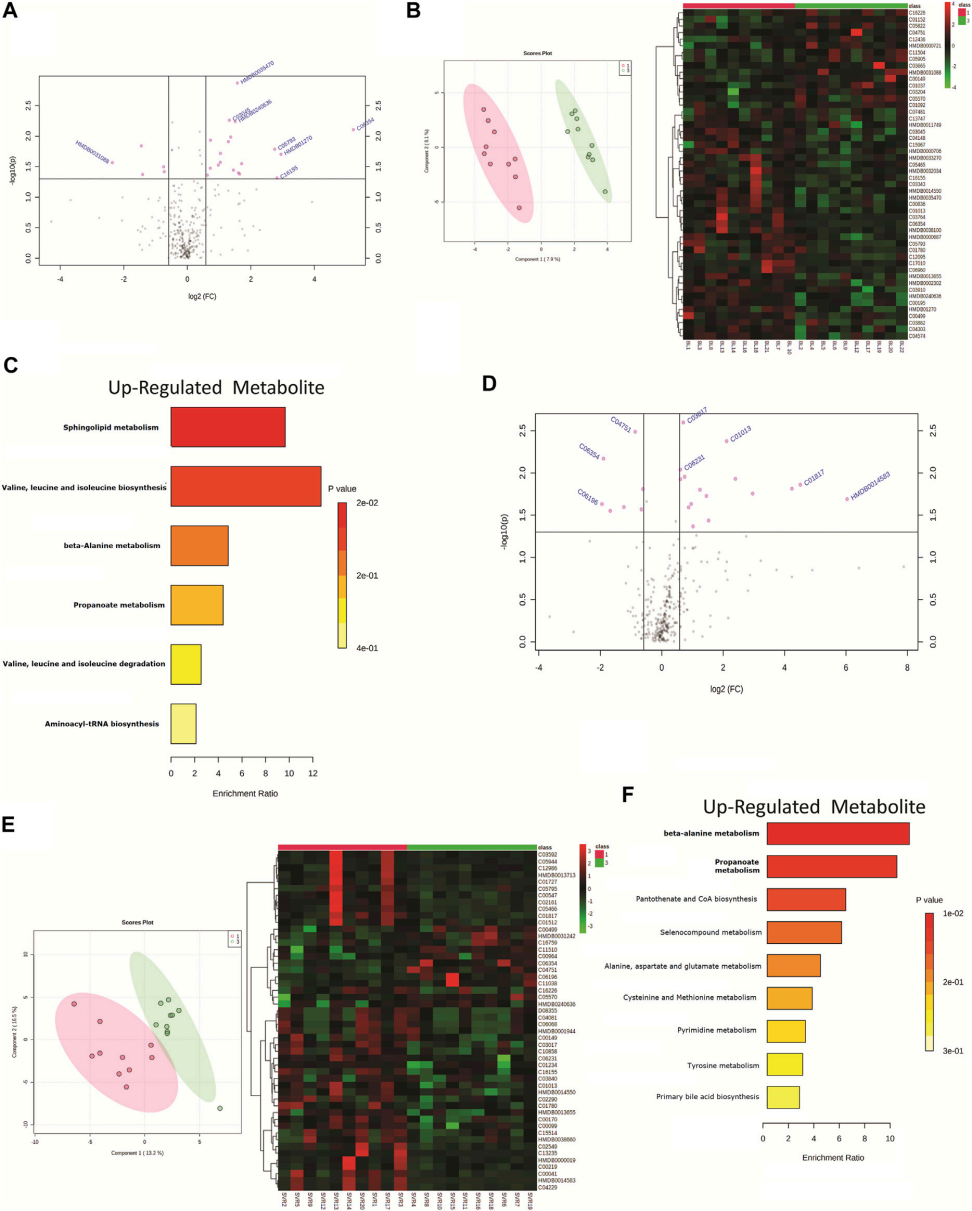
**(A)** Volcano plot showing differentially expressed metabolites in patients infected with genotype 1 HCV compared to genotype 3 HCV. Pink dots are significant at baseline (*p* < 0.05). Pink dotes corresponds to non-responders and green dots corresponds to responders. **(B)** Partial least square discriminant analysis (PLSDA) and Heat map and hierarchical cluster showing clear segregation of patients infected with genotype 1 HCV compared to genotype 3 HCV, at baseline based on the metabolome profile of patients. **(C)** KEGG pathway analysis of up regulated metabolites in patients infected with genotype 1 HCV compared to genotype 3 patients at baseline. **(D)** Volcano plot showing differentially expressed metabolites in patients infected with genotype 1 HCV compared to genotype 3 HCV post therapy. Pink dots are significant at (*p* < 0.05). **(E)** Partial least square discriminant analysis (PLSDA) and Heat map and hierarchical cluster showing clear segregation of patients infected with genotype 1 HCV compared to genotype 3 HCV, Post therapy based on the metabolome profile of patients. **(F)** KEGG pathway analysis of up regulated metabolites in patients infected with genotype 1 HCV compared to genotype 3 patients post therapy.

The HIV–HCV patients included in the study were HCV genotype 1 (*n* = 10) or genotype 3 (*n* = 10). A total of 24 differentially expressed metabolites (DEMs; (FC > 1.5, *p* < 0.05)) were identified when the baseline metabolite profile of HCV genotype 1 was compared to that of HCV genotype 3 (Figure 3A, Supplementary Table S2). Partial least square discriminant analysis and hierarchical clustering analysis segregated HCV genotype 1 from genotype 3 based on the baseline metabolome profile (Figure 3B). Of those, 19 DEMs were significantly upregulated and linked to sphingolipid metabolism, isoleucine, leucine, valine metabolism, and others (Figure 3C). A total of 5 metabolites were downregulated in HCV genotype 1 but did not significantly enrich any pathways. Further analysis of post-DAA therapy plasma metabolomes for patients infected with genotype 1 compared to those of genotype 3 identified 23 differentially expressed metabolites (DEMs; FC > 1.5, *p* < 0.05) (Figure 3D, Supplementary Table S3). Partial least square discriminant analysis and hierarchical clustering analysis segregated HCV genotype 1 from genotype 3 based on the baseline metabolome profile (Figure 3E). Of those, 16 were upregulated and associated with beta-alanine metabolism, propionate metabolism, and others (Figure 3F, *p* < 0.05, FC > 1.5, Supplementary Table S2). A total of 7 metabolites were downregulated but did not yield any significant pathway (*p* < 0.05, FC > 1.5, Supplementary Table S2). These results suggest that the HCV genotype is associated with the plasma metabotype and change in the genotype also alters the metabotype, and its association with DAA-nonresponse may require further analysis.

### Identification of a Panel of a Metabolite That Can Differentiate DAA Nonresponders From Responders at Baseline

**FIGURE 4:**
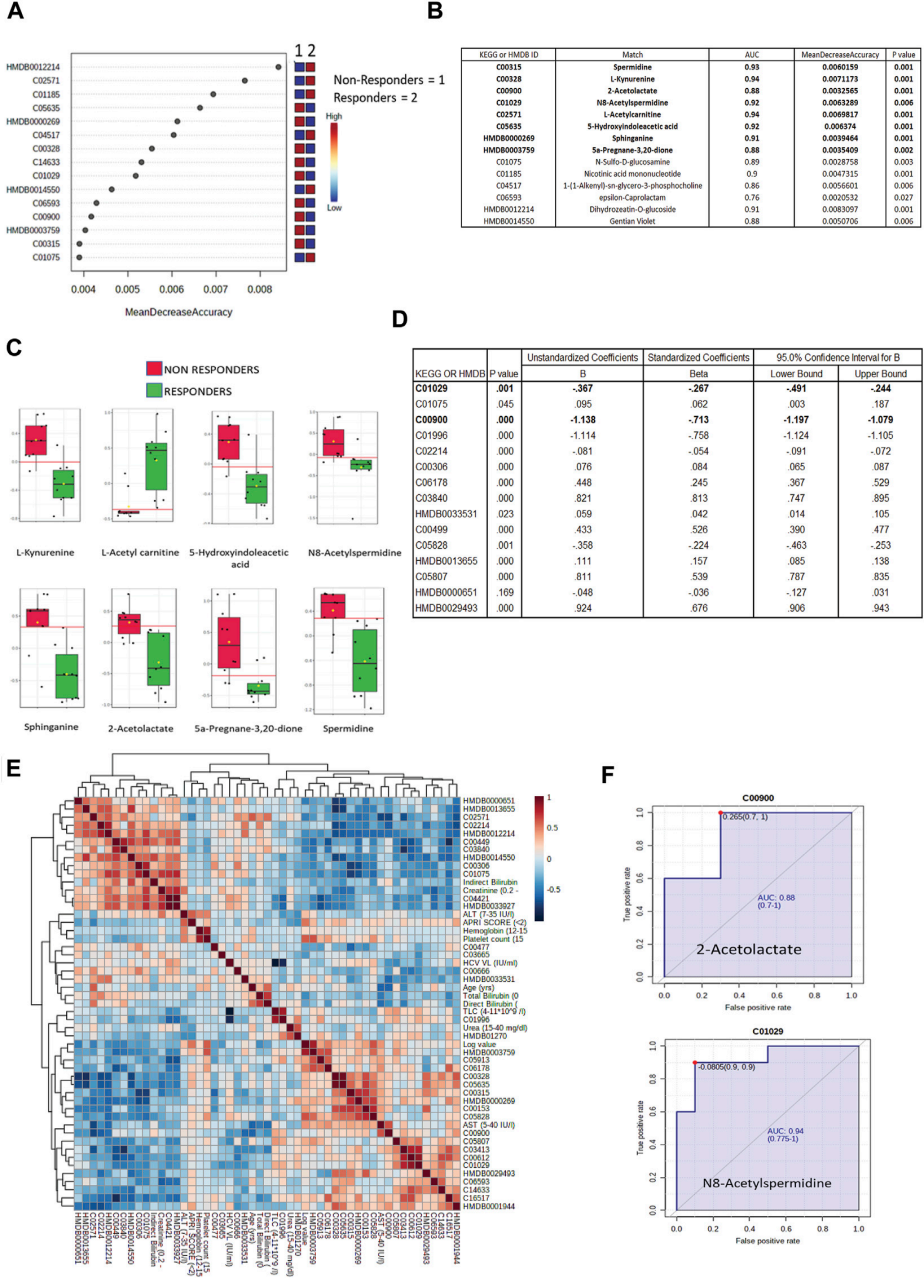
**(A)** Mean decrease in accuracy plot showing the mean decrease in accuracy of the metabolites along with their expression status Red = upregulated and blue = downregulated in DAA Non responders as compared to in DAA responders. **(B)** AUC and mean decrease accuracy of metabolites in DAA Non responders as compared to in DAA responders at baseline. **(C)** Relative abundance (Log normalized) for metabolites showing significant difference In DAA Non-responders and DAA Responders at baseline based on the metabolome profile of patients. **(D)** Multivariate linear discriminating analysis documenting Beta factor for all the metabolites significantly associated with DAA response at baseline. **(E)** Correlation plot of important metabolites and clinical features. **(F)** AUROC analysis of 2-Acetolactate and N8-Acetyl Spermidine.

All differentially expressed metabolites at baseline between DAA nonresponders and responders were subjected to a random forest analysis, and the top 15 significant metabolites with minimum mean decrease accuracy and AUC value ranging between 0.76–0.94 were identified (Figures 4A,B). Some important metabolites such as L-kynurenine, 5-hydroxyindoleaceticacid, N8-acetylspermidine, sphinganine, spermidine, and 2-acetolactate were found upregulated in DAA nonresponders (Figure 4C, Supplementary Figure S10). Baseline differentially expressed metabolites (*n* = 39) were subjected to univariate and multivariate linear regression analysis, which resulted in the identification of a panel of 15 metabolites linked to nonresponse to DAA therapy in HIV–HCV patients (Figure 4D). Considering random forest and multivariate regression analysis, metabolites such as 2-acetolactate (AUROC > 0.8) and N8-acetylspermidine (AUROC > 0.8; Figure 4F) were identified as the most significant metabolite which can differentiate DAA nonresponders from potential responders even at baseline. Finally, correlation analysis showed that both 2-acetolactate and N8-acetylspermidine showed a direct correlation with AST, log HCV count, urea content, TLC, and APRI score (r2 > 0.5; *p* < 0.05) (Figure 4E).

## Discussion

In the present study, the plasma metabolome profile of HIV–HCV patients on DAA therapy was evaluated and compared between the responders and nonresponders. Plasma metabolome analysis was performed at baseline and post 3 months of DAA therapy in HIV–HCV coinfected patients. The primary aim of the analysis was to identify baseline metabolites that could identify potential nonresponders of the DAA therapy. We wanted to identify the metabolic difference in HCV nonresponders and responders at baseline and compare them to the post-therapy metabolome profile. Furthermore, the HCV genotype associated with the plasma metabolomics was also documented in the study. Amongst all the differentially expressed metabolites, the mean decrease in the accuracy (calculated by random forest; 2,000 trees) was high for metabolites such as L-kynurenine, 5-hydroxyindoleaceticacid, N8-acetylspermidine, sphinganine, spermidine, and 2-acetolactate. Furthermore, AUROC combined with multivariate linear regression analysis ultimately identified baseline levels of N-acetylspermidine (AUC = 0.940) and 2-acetolactate (AUC = 0.880) and can be validated in a larger cohort of HIV–HCV patients for their ability to predict the response of the patient toward DAA therapy.

To identify the difference in DAA therapy responders and nonresponders, a total of 20 paired HIV–HCV patient samples were identified, and their baseline and post-therapy (3 months) plasma samples were subjected to metabolomic evaluations. Based on the post-therapy HCV RNA status, these patients were further segregated as responders (*n* = 10) and nonresponders (*n* = 10) to standard HCV therapy. Clinically, the responders and nonresponders were identical except for post-therapy HCV log values, which were significantly different in nonresponders compared to responders (*p* < 0.05). We also observed a significant difference in their plasma metabolome profile. Around 39 differentially expressed metabolites (DEMs) were identified and subjected to pathway analysis in our study. The most important pathway that got significantly altered in DAA nonresponders compared to those in responders was Tryptophan metabolism. The tryptophan to kynurenine ratio is already suggested by Kardashian et al. (Kardashian et al., 2019) as a predictor of liver progression toward fibrosis. Identification of tryptophan-linked metabolites (kynurenine and 5-hydroxyindoleacetic acid) even in our non-cirrhotic cohort, at first, validates our metabolome analysis and provides a basis that alteration of tryptophan metabolites correlates with liver injury and associated immune activation. Further identification of these metabolites or their ratio could be exploited to understand the complexity of HCV infection, particularly fibrosis in HIV–HCV coinfected patients.

**FIGURE 5:**
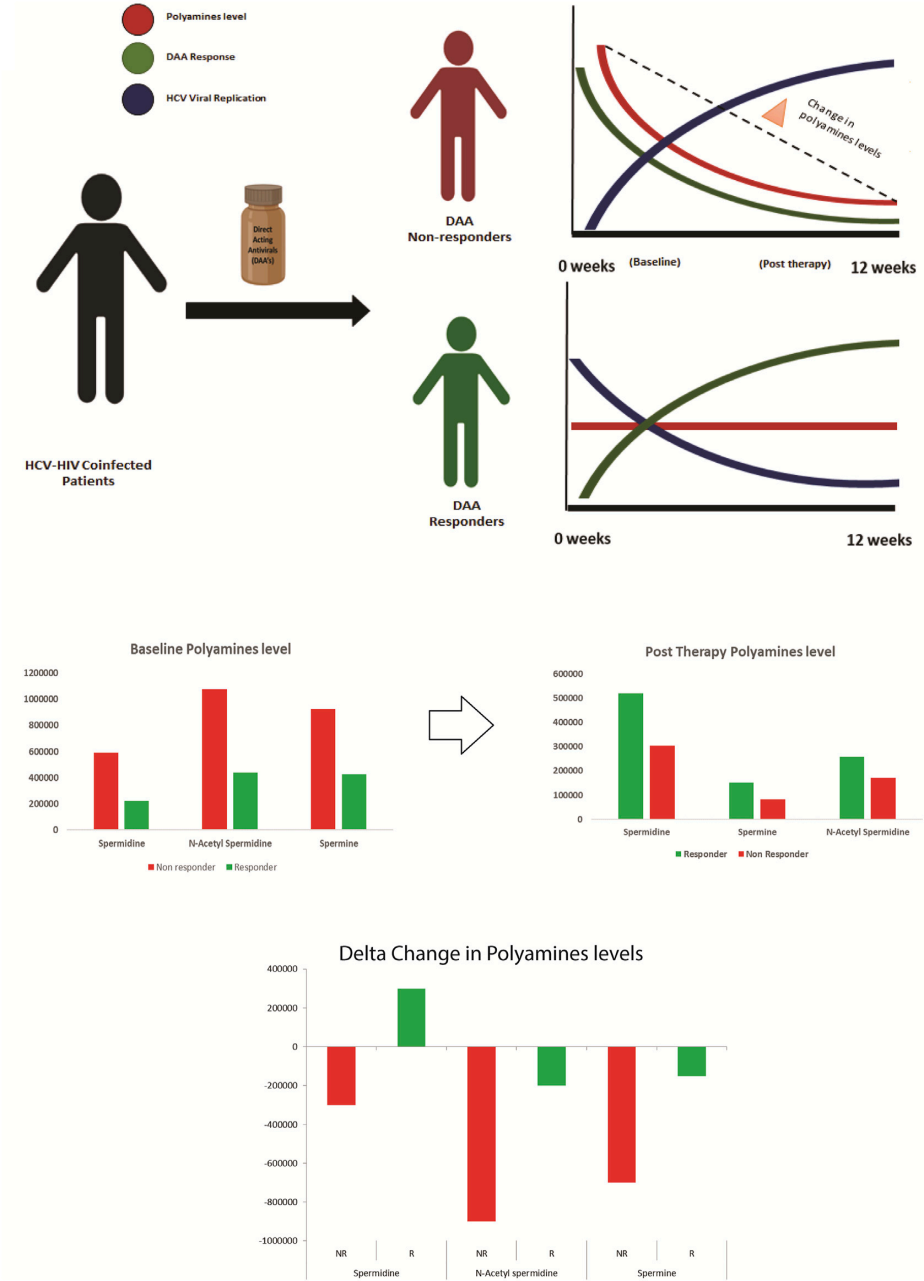
Paradigm of DAA non Response: Metabolomics analysis shows that decrease in polyamines from baseline to post therapy correlates with the increase in viral replication and DAA non response. Thus baseline measurement of polyamines is crucial for segregating patients predisposed to DAA non response.

Analysis of the post-DAA therapy plasma metabolome profile in DAA nonresponders compared to that in DAA responders showed that the nonresponders have decreased levels of tryptophan, cytosine, L-histidine, indoacrylic acid, 3-methyl indole, and others and were associated with pathways such as tryptophan metabolism, pyruvate metabolism, lipids metabolism, valine, isoleucine, and leucine metabolism, and others. Comparative analysis of the baseline and post-therapy metabolome profile of DAA nonresponders highlighted a significant reduction of tryptophan metabolism in the post-therapy samples compared to baseline line metabolomic alterations. Pathways significantly upregulated in nonresponders at baseline include arginine and proline metabolism, alanine metabolism, sphingolipid metabolism, nicotinamide metabolism, and others. In comparison, post-therapy plasma samples documented significant downregulation of pathways linked to pyruvate metabolism, lipids metabolism, valine, isoleucine, and leucine metabolism, and others in nonresponders compared to responders. This suggests that polyamines play a vital role in HCV–HIV coinfected individuals (Mounce et al., 2017). An increase in the expression of polyamines seen explicitly in the nonresponders boosts the viral life cycle, thereby negating the effect of DAA or other antivirals (Figure 5).

In detail, polyamines are flexible amino group carrier carbon compounds that are positively charged at cellular pH (Casero and Woster, 2009). Key metabolites involved in polyamine biosynthesis are arginine, ornithine, putrescine, spermidine, spermine, etc. (Pegg, 2009). Polyamine synthesis is an energetically expensive process for cells; thus, their regulation is very tightly controlled, and this regulation is majorly performed by polyamines themselves (Bekebrede et al., 2020). As they are positively charged, they affect chromatin’s condensation and thus can modulate differential gene expression (Firpo and Mounce, 2020).

Polyamines play a vital role in viral life cycles, ranging from viral entry, viral replication, viral transcription, viral translation, chromatin modifications, and virion packaging (Firpo and Mounce, 2020). In viral infections such as HCV, flaviviruses Zika virus (ZIKV), and chikungunya virus (CHIKV), polyamines enhance viral polymerase activity (Firpo and Mounce, 2020). Recent studies proved that HCV enhances polyamine production, which supports enhanced viral replication, thus ultimately depleting their level during infection (Smirnova et al., 2017).

Metabolomics analysis of the plasma captures this phenomenon and shows that polyamines are depleted in post-therapy samples, specifically in nonresponders. This decrease in the polyamine level post therapy in the nonresponders could be attributed to a significant increase in the HCV viral replication observed in such patients (Smirnova et al., 2017).

Our analysis also showed that the plasma metabolome profile of HCV genotype 1 patients is different from that of HCV genotype 3 patients both at baseline and after 3 months of DAA therapy. At baseline, HCV genotype 1 has significantly higher levels of metabolites linked to sphingolipid metabolism, B-alanine metabolism, and others. Post-therapy metabolites linked to B-alanine metabolism, propionate metabolism, pantothenate, and seleno compounds were increased in HCV genotype 1 patients in the HIV–HCV cohort. This observed change in the metabolome profile between genotype 1 and 3 could also be associated with nonresponse to DAA therapy, though this observation requires further validation.

Finally, amongst all the differentially expressed metabolites at baseline, the mean decrease in the accuracy (calculated by random forest; 2,000 trees) was the highest for metabolites such as L-kynurenine, 5-hydroxyindoleaceticacid, N8- acetylspermidine, sphinganine, spermidine, 2-acetolactate, and others. Combining random forest and multivariate linear regression analysis, we identified N-acetyl spermidine (AUC = 0.940, B factor = −0.267) and 2-acetolactate (AUC = 0.880, B factor = −0.713), which were significantly increased and were capable to segregate DAA nonresponders from responders in HIV–HCV coinfected patients at baseline. N8-acetylspermidine is a polyamine (Mounce et al., 2017), and 2-acetolactate is reported to be altered in Type 2 diabetes (Li et al., 2019). A significant increase in these metabolites in DAA nonresponders for HCV in HIV–HCV patients is unique and thus could be validated in a bigger cohort to identify their potential as a candidate baseline indicator of response in DAA therapy for HCV in HIV–HCV coinfected patients.

## Data Availability

The original contributions presented in the study are included in the article/Supplementary Material; further inquiries can be directed to the corresponding authors.

## Abbreviations

DAA, direct-acting antivirals; HCV, hepatitis c virus; HIV, human immunodeficiency virus; PWID, patients with intravenous drug abuse; SVR, sustained viral response; AUC, area under the ROC curve; MSM, men who had unprotected sex with other males; ATMH, antiretroviral therapy–mediated hepatotoxicity; PEG-IFN, pegylated interferon; RBV, ribavirin; HRMS, high-resolution orbitrap mass spectrometry.
